# Trajectory log analysis and cone‐beam CT‐based daily dose calculation to investigate the dosimetric accuracy of intensity‐modulated radiotherapy for gynecologic cancer

**DOI:** 10.1002/acm2.13163

**Published:** 2021-01-10

**Authors:** Yohei Utena, Jun Takatsu, Satoru Sugimoto, Keisuke Sasai

**Affiliations:** ^1^ Department of Radiation Oncology Graduate School of Medicine Juntendo University Tokyo Japan; ^2^ Department of Radiology Toranomon Hospital Tokyo Japan; ^3^ Department of Radiation Oncology Faculty of Medicine Juntendo University Tokyo Japan

**Keywords:** CBCT‐based dose calculation, delivery verification, geometric uncertainties, IMRT, log file analysis

## Abstract

This study evaluated unexpected dosimetric errors caused by machine control accuracy, patient setup errors, and patient weight changes/internal organ deformations. Trajectory log files for 13 gynecologic plans with seven‐ or nine‐beam dynamic multileaf collimator (MLC) intensity‐modulated radiation therapy (IMRT), and differences between expected and actual MLC positions and MUs were evaluated. Effects of patient setup errors on dosimetry were estimated by in‐house software. To simulate residual patient setup errors after image‐guided patient repositioning, planned dose distributions were recalculated (blurred dose) after the positions were randomly moved in three dimensions 0–2 mm (translation) and 0°–2° (rotation) 28 times per patient. Differences between planned and blurred doses in the clinical target volume (CTV) D_98%_ and D_2%_ were evaluated. Daily delivered doses were calculated from cone‐beam computed tomography by the Hounsfield unit‐to‐density conversion method. Fractional and accumulated dose differences between original plans and actual delivery were evaluated by CTV D_98%_ and D_2%_. The significance of accumulated doses was tested by the paired t test. Trajectory log file analysis showed that MLC positional errors were −0.01 ± 0.02 mm and MU delivery errors were 0.10 ± 0.10 MU. Differences in CTV D_98%_ and D_2%_ were <0.5% for simulated patient setup errors. Differences in CTV D_98%_ and D_2%_ were 2.4% or less between the fractional planned and delivered doses, but were 1.7% or less for the accumulated dose. Dosimetric errors were primarily caused by patient weight changes and internal organ deformation in gynecologic radiation therapy.

## 1 INTRODUCTION

Intensity‐modulated radiotherapy (IMRT) techniques are capable of creating highly conformal dose distributions with sparing of normal tissues. However, a previous study reported that the steep dose distribution led to large dosimetric errors caused by several patient setup errors.^1^ Therefore, an accurate setup is necessary.

To verify accurate patient setup, image‐guided radiation therapy (IGRT) with cone‐beam computed tomography (CBCT) is an essential technique. Patient setup with three‐dimensional matching using kV‐CBCT images improves the repeatability of patient positioning as compared with conventional two‐dimensional matching.^2^ However, the matching of planning CT and CBCT images is performed on the assumption that there are no anatomical changes. In general, patient weight changes, tumor shrinkage, and deformation of the internal organs occur during the treatment period over 1 month. In particular, the daily variations of bladder and rectum filling must be considered for radiation therapy of the pelvic region.

Many studies have proposed methods for evaluation of the dosimetric effect of anatomical changes on the dose of the treatment day by using the deformable image registration (DIR) technique.^3^, ^4^, ^5^, ^6^ Although the DIR technique enables assessment of the dosimetric effect of internal organ deformations, variations in patient weight are not considered when assessing the effect on the dose of the day.^7^ In addition, low contrast and artifacts of CBCT images cause inaccuracies in DIR.^8^

In this study, we investigated dose calculations by using CBCT images to evaluate the dosimetric effect of three factors: mechanical control accuracy of the treatment machine, patient setup errors, and interfractional geometric variations of target and other structures. We classified the factors related to dosimetric differences between planned doses and delivered doses into three categories and quantitatively evaluated their effect on delivered doses.

## 2 MATERIALS AND METHODS

### 2.A Patient population

Thirteen consecutive patients who underwent IMRT of the whole pelvis to treat cervical carcinoma at our institution were selected. This retrospective study was approved by the ethics committee of our institution.

### 2.B Contouring

To improve the precision of the patient positioning reproducibility, knees and ankles were fixed with a Vac‐Lok positioning bag (CIVCO, Kalona, IA) for all patients. Planning computed tomography (CT) images were acquired with a 3‐mm slice thickness (Toshiba Aquilion LB, Canon Medical Systems, Ōtawara, Japan). All patients were instructed to empty their bladders and rectums, but two were instructed to drink 200 mL of water 1 hour before CT scans and each treatment to spare the small bowel.

The clinical target volume (CTV) was defined as all areas of primary tumor and regional lymph nodes by an experienced radiation oncologist. The delineations of pelvic lymph nodes were followed by the guidelines of the Japan Clinical Oncology Group Gynecologic Cancer Study Group.^9^ The internal target volume of the cervix was defined as the volume expanded 15 mm in all directions, excluding bones. Then, the planning target volume (PTV) margin of 7 mm in all directions around the CTV was added to take into account setup errors and uncertainties of inter/intrafractional organ motions. The details of contouring CTVs and organs at risk (OARs) were reported in Ref. [10].

### 2.C Treatment planning

All patients were planned by using an Eclipse version 13.6 (Varian Medical Systems, Palo Alto, CA) and an analytical anisotropic algorithm. The resolution used for dose calculation was 2 mm in all directions. IMRT plans were optimized on a seven‐ or nine‐beam dynamic multileaf collimator (DMLC) delivery system using a 10‐MV photon beam produced by a TrueBeam linear accelerator with a Millenium 120 MLC (Varian Medical Systems). The maximum speed of leaf motion is 2.5 cm/s. All plans were carried out with fixed jaw technique which keeps jaws in the same position during irradiation. The dose prescribed to the PTV was 50.4 Gy in 28 daily fractions. The planning goal was to achieve 50% or more of the PTV receiving the prescription dose, 95% of the PTV receiving 98% of the prescription dose, and then 0% of the PTV receiving 110% of the prescription dose. In addition, dose constraints for OARs were followed by the JCOG 1402 protocol.^11^

### 2.D Evaluation of mechanical control accuracy

To evaluate the uncertainties in mechanical control, trajectory log files were analyzed. The log file includes planned and recorded machine parameters of MLC positions, gantry angle, jaw positions, and accumulated monitor units (MUs). The time resolution of recorded parameters for the log file of TrueBeam is 20 ms. In this study, differences in the actual MLC positions and delivered MUs from planned parameters were analyzed for all of the recorded data. Log files of the first fraction were analyzed for all patients.

Plan parameters of all IMRT plans were calculated by using in‐house software developed using the Eclipse scripting application programming interface (API), version 13.6. The mean aperture size and mean MLC gap width were calculated from the leaf positions for each control point.

### 2.E Simulations of dosimetric uncertainties caused by patient setup errors

The simulation method was based on stochastic properties of rigid motions.^12^ To investigate dosimetric uncertainties caused by patient setup errors, in‐house software written by the Eclipse scripting API was used to perform the simulations. This study simulated residual setup errors after patient repositioning for CBCT‐based IGRT. Planning CT was used to evaluate the effect of pure setup error without consideration of patient weight changes on dose delivery to CTV. First, in‐house software blurred the planned doses for rotational and translational setup errors in three dimensions. Previous study reported that patient setup errors after repositioning with a six degrees of freedom (6DOF) couch were 1.6±0.8 mm.^13^ Therefore, planned doses were randomly blurred from 0 to 2 mm in translation and from 0° to 2° in rotation and simulated 28 times per patient. In this work, dose blurring means that the isodose cloud was randomly blurred. Therefore, this study did not consider inhomogeneity correction. Second, the maximum deviations in the CTV D_98%_ and D_2%_ between the planned doses and blurred doses were evaluated.

### 2.F CBCT‐based dose calculation

All patients underwent CBCT scans prior to every treatment. The Varian On‐board Imager Spotlight protocol was used.^14^ The field of view (FOV) was 26 cm in diameter. CBCT scans were matched onto the planning CT scan according to the bony anatomy by using 3D/3D matching. From the results of the 3D/3D matching, a patient was repositioned on a PerfectPitch 6DOF couch (Varian Medical Systems). To evaluate dosimetric effects caused by variations in the patient’s weight and deformations of the internal organs, CBCT‐based dose calculations were performed by using PerFRACTION ver. 2.3.4 (SunNuclear, Melbourne, FL). The CBCT images with a small FOV did not encompass the entire body outline, so the planning CT images were used for the outside of the FOV with an online registration matrix. To consider the variations in patient weight, dose calculations were performed within 2 cm outside of the body outline. Dose calculations were performed with each Hounsfield unit (HU)‐to‐density curve for planning CT images and CBCT images. CBCT‐based delivered dose calculations of 358 images in 13 patients were compared with the planned doses. Six fractions with data corruption were excluded for dosimetric analysis. To remove the difference in the dose calculation algorithm from the analysis results, the planned dose was also recalculated according to the Collapsed‐Cone Convolution/Superposition algorithm of PerFRACTION. The dose grid was set to the same resolution as that of the treatment planning system (2 mm) in all directions. CBCT‐based delivered doses were compared with the planned doses for each treatment day and the accumulated doses for the entire treatment.

Moreover, there has been no report comparing the planned dose and a deformed accumulation dose of CBCT‐based dose calculation for all fractions in the pelvic region. The purpose of this study was to evaluate the deformed dose with commercial software without performing any special image processing. Then, the deformed accumulated dose was evaluated only in patients who had small interfractional organ motion, CBCT images had no artifacts, and were able to contour OARs (bladder, rectum, and femoral heads) on CBCT images for all fractions. Fractional delivered dose was deformed to planning CT using deformed vector field (DVF) generated with RayStation ver.9.A (RaySearch Laboratories, Stockholm, Sweden). In this study, hybrid DIR algorithm that uses both intensity‐based and structure‐based DIR was used.^15^ The online 3D/3D matching was used for an initial rigid registration. Deformation accuracy was evaluated using dice similarity coefficients (DSCs) of bladder, rectum, and femoral heads.^16^ DSC measures the overlap volume between the ROI contoured by planning CT and the ROI deformed from CBCT to planning CT. DSC is widely used to evaluate deformation accuracy. Finally, the deformed fractional delivered dose was accumulated. Deformed accumulated doses were compared with the planned doses for D_98%_ and D_2%_ of CTV and D_mean_ of OARs.

For dose comparisons of planned doses and CBCT‐based delivered doses, Student’s paired t test was used with R version 3.6.0 software (R Foundation, Vienna, Austria). Statistical significance was set at the 5% level, *P* < 0.05.

## 3 RESULTS

### 3.A Evaluation of mechanical control accuracy

From log file analysis, the MLC positional errors and MU delivery errors were −0.01 ± 0.02 mm and 0.10 ± 0.10 MU, respectively. The mechanical accuracy of TrueBeam was found to be well below the recommended tolerances by AAPM TG142.^17^ From the analysis of plan parameters, the mean aperture size, mean differences in MLC gap width, and mean total MU were 34.4 ± 20.5 cm^2^, 16.9 ± 9.9 mm, and 1528 ± 117 MU, respectively.

### 3.B Simulations of dosimetric uncertainties for patient setup errors

Table 1 shows the maximum differences between the blurred dose and planned dose in each patient. For all patients, the differences in the DVH parameters (D_98%_ and D_2%_) from the original plan were 0.5% or less. Residual setup errors ≤2 mm and 2° did not affect the target coverage.

**Table 1:** Comparison of DVH parameters (D_98%_ and D_2%_) between the blurred dose and planned dose.

Patient	ΔD_98%_ (planned ‐ blurred)	ΔD_2%_ (planned ‐ blurred)
1	−0.5%	0.0%
2	−0.2%	0.0%
3	0.0%	0.0%
4	0.5%	−0.1%
5	0.0%	−0.3%
6	0.0%	−0.5%
7	−0.5%	−0.2%
8	0.5%	0.0%
9	0.0%	0.1%
10	0.0%	−0.2%
11	0.0%	−0.3%
12	0.1%	−0.3%
13	0.0%	0.2%

### 3.C Evaluation of fractional CBCT‐based delivered dose

**Fig. 1:**
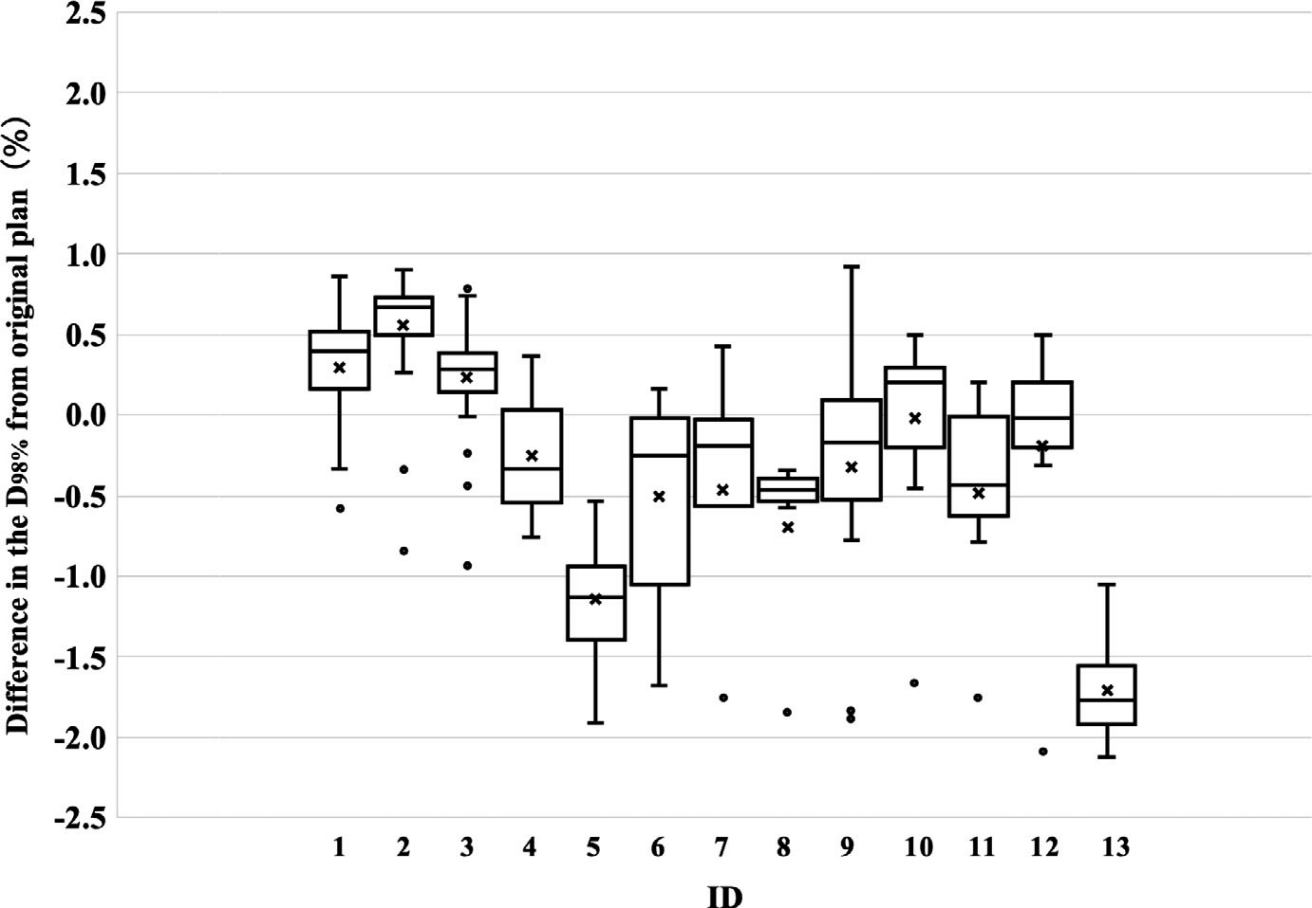
Box and whisker plots of the relative dose differences in the clinical target volume D_98%_ between planned doses and cone‐beam computed tomography‐based fractional doses. The central solid line and cross indicate the median value and mean value, respectively. The borders of the box indicate the 25th and 75th percentiles. The outliers are plotted as dots.

**Fig. 2:**
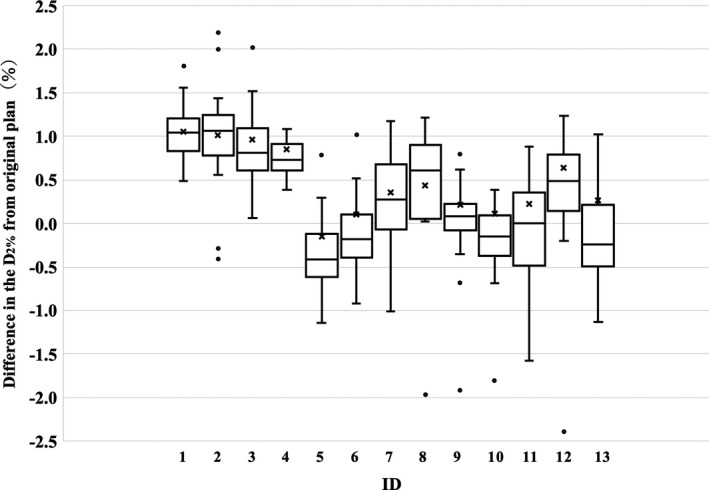
Box and whisker plots of the relative dose differences in the clinical target volume D_2%_ between the planned doses and cone‐beam computed tomography‐based fractional doses. The central solid line and cross indicate the median value and mean value, respectively. The borders of the box indicate the 25th and 75th percentiles. The outliers are plotted as dots.

To investigate the effect of interfractional rigid or nonrigid geometric variations on the CTV dose coverage, the planned dose and CBCT‐based fractional delivered doses were compared. Box and whisker plots of the relative dose differences in the CTV D_98%_ and D_2%_ for each treatment day are shown in Figs. 1 and 2. Differences in CTV dose coverages were within 2.5% on all treatment day for all patients. However, as evident in Fig. 1, patient 2 received systematic dose increases, and patients 5 and 13 received systematic dose reductions.

### 3.D Evaluation of accumulated CBCT‐based delivered dose

**Fig. 3:**
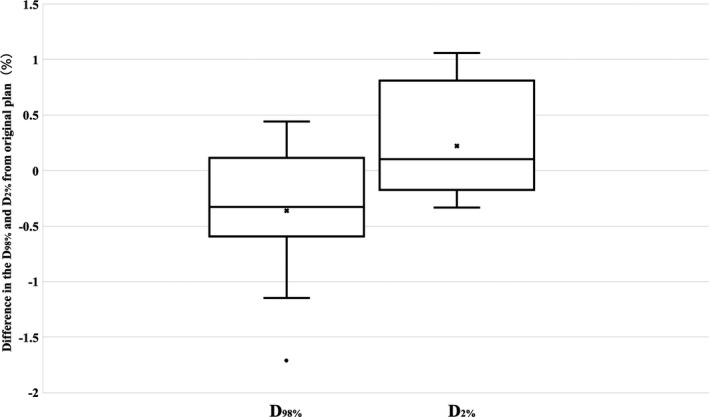
Box and whisker plots of the relative dose differences in the clinical target volume D_98%_ and D_2%_ between planned doses and cone‐beam computed tomography‐based accumulated doses. The central solid line and cross indicate the median value and mean value, respectively. The borders of the box indicate the 25th and 75th percentiles. The outliers are plotted as dots.

Figure 3 demonstrates box and whisker plots of dose differences in the CTV D_98%_ and D_2%_ between the planned and accumulated delivered doses. Although the relative differences in the fractional delivered dose were 2.4% or less, those in the accumulated delivered doses decreased to 1.7% or less for both the CTV D_98%_ and D_2%_. No significant differences in D_98%_ (*P* = 0.051) and D_2%_ (*P* = 0.132) between the planned doses and delivered doses were observed.

After the visual assessments, DIR was performed on 2 to 13 patients who were able to be contoured without artifacts in all fractions (patients 2 and 8). Table 2 shows the DSCs of bladder, rectum and right/left femoral head. Except for the patient 8 bladder, the DSCs exceeded 0.8, which was a sufficient deformation accuracy.^18^ Bladder of patient 8 had low DSC in some fractions due to interactional volume variations.

**Table 2:** Dice similarity coefficients of bladder, rectum, and femoral heads for patients 2 and 8.

	Bladder	Rectum	Femoral head (R)	Femoral head (L)
Patient 2	0.95 ± 0.02	0.92 ± 0.16	0.99 ± 0.01	0.99 ± 0.01
Patient 8	0.67 ± 0.24	0.80 ± 0.08	0.98 ± 0.01	0.99 ± 0.01

Abbreviation: Femoral head (R): right femoral head; Femoral head (L): left femoral head

**Fig. 4:**
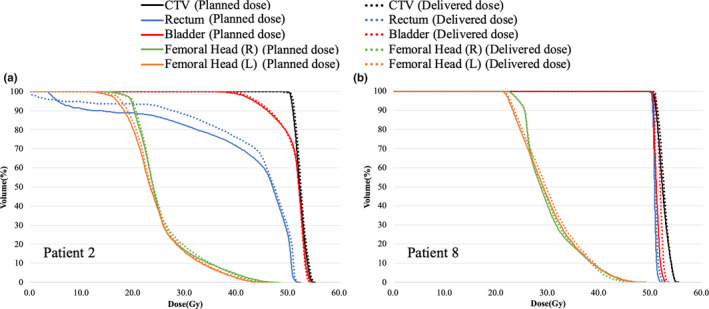
Dose‐volume histograms of clinical target volume, rectum, bladder, and femoral head (R, L) for patient 2 (a) and patient 8 (b). Solid line is the planned dose calculated on planning computed tomography (CT) image, and dashed line is the delivered dose calculated on cone‐beam CT images.

Table 3 shows comparison of DVH parameters for the CTV, bladder, rectum, and femoral heads between the deformed accumulation doses and the planned doses for patients 2 and 8. In Fig. 3, dose difference in the CTV D_98%_ and D_2%_ between the accumulated delivered doses and the planned doses were within 1% for both patients 2 and 8. Dose difference in the CTV D_98%_ and D_2%_ between the deformed accumulation doses and the planned doses were also within 1%. However, the dose difference of rectum in patient 2 showed the large difference (4.6%). In addition, DVH curves of patients 2 and 8 are shown in Fig. 4. In patient 8, the DVH curve was consistent between the two doses, and the DVH parameters were also consistent within 2% including OARs. In contrast to patient 8, patient 2 showed the inconsistency of DVH curve between the two doses.

**Table 3:** Comparison of DVH parameters between the deformed accumulation dose and the planned dose for patients 2 and 8.

	ΔD_98%_ (planned ‐ deformed)	ΔD_2%_ (planned ‐ deformed)	ΔD_mean_ (planned ‐ deformed)			
	CTV		Bladder	Rectum	Femoral head (R)	Femoral head (L)
Patient 2	0.5%	0.6%	0.1%	4.6%	0.9%	1.6%
Patient 8	0.6%	0.0%	1.3%	0.5%	0.4%	1.4%

### 3.E Effects of patient weight changes and internal organ deformations

**Fig. 5:**
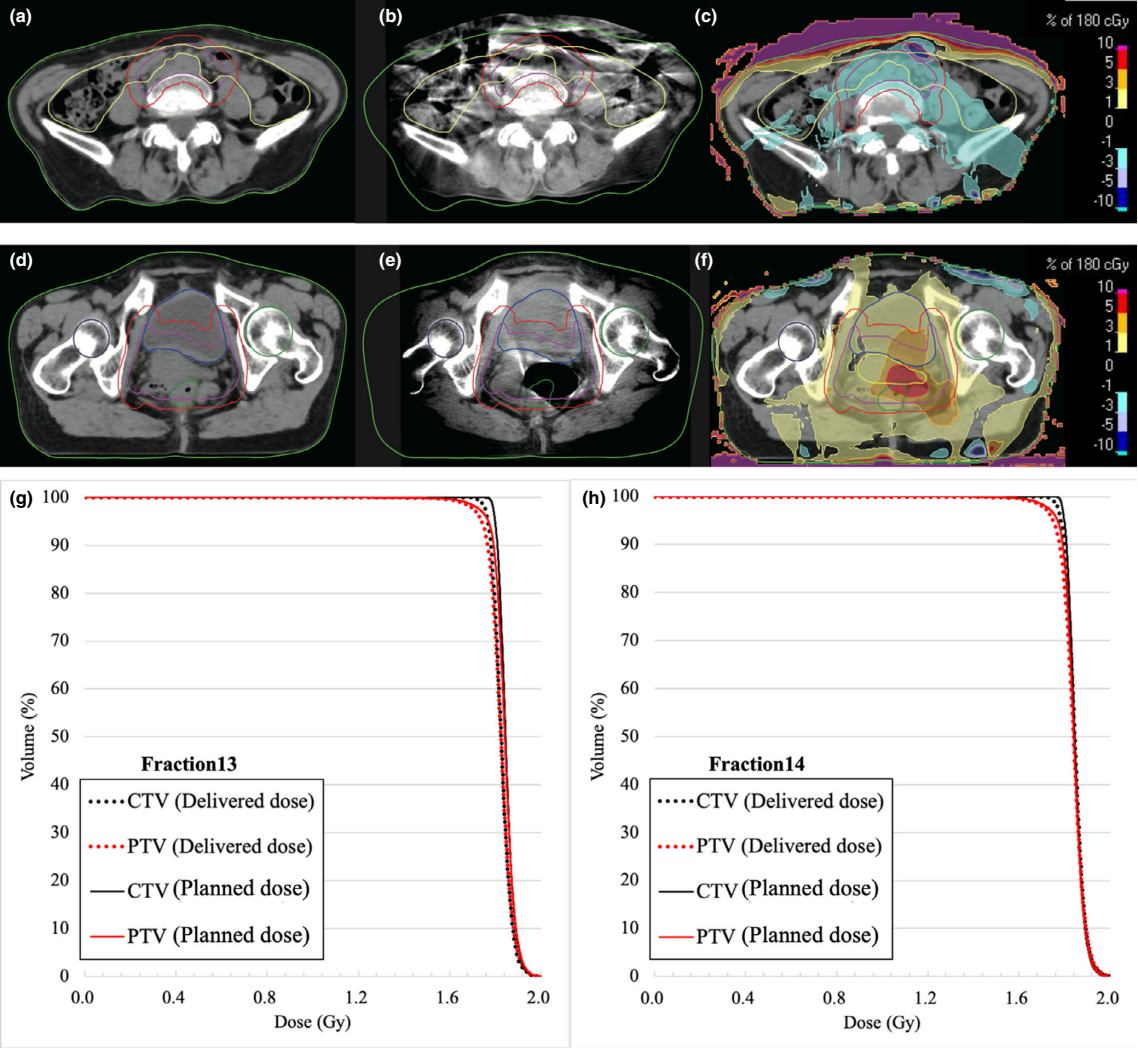
Example of body weight changes for patient 13. (a) Planning computed tomography (CT) image for fraction 13, (b) cone‐beam CT image for fraction 13, and (c) dose differences between the fractional planned and delivered dose. Example of anatomical changes for same patient, 13. (d) Planning CT image for fraction 14. (e) CBCT image for fraction 14. (f) Dose differences between the fractional planned and delivered dose. (a)−(c) and (d)−(f) show the same planes. Dose‐volume histograms of patient 13 for fraction 13 (g) and fraction 14 (h). Solid line is the fractional planned dose calculated on planning CT image, and dashed line is the fractional delivered dose calculated on CBCT images.

Figure 5 shows a comparison of planning CT, CBCT images, and dose difference for patient 13 on fractions 13 and 14. The skin surface changed over 1.5 cm from planning CT to CBCT image on fraction 13 in Figs. 5(a) and 5(b). The bigger belly size on fraction 13 than that on planning CT represent large dose decrease in the CTV.

Figures 5(d)−5(f) shows an example of dose increase due to the presence of gas pockets. As shown in Fig. 4(e), gas pocket that did not exist at the time of planning CT was present on fraction 14. Although the presence of the gas pocket caused over 5% dose increases in the CTV and gas pocket, difference of CTV D2 between planned dose and fractional delivered dose on fraction 14 was within 0.4%. Furthermore, Figs. 5(g) and 5(h) show DVH curves of patient 13 for fractions 13 and 14, respectively. Dose decreases caused by the patient weight change for fraction 13 and dose increases caused by gas pocket for fraction 14 were also seen in DVH curves.

## 4 DISCUSSION

In this study, dosimetric uncertainties in radiation therapy were classified into three components and quantitatively evaluated. The log file analysis results represent high accuracy of delivery parameters, which includes leaf position and dose rate. Previous studies have reported high mechanical accuracy of TrueBeam used with the VMAT technique,^19^, ^20^ and the results of those studies are consistent with those of the present study. In contrast to the VMAT technique, the DMLC technique does not need to consider uncertainties regarding gantry angle positioning. Rangel et al. reported that dose differences caused by leaf positional errors for head‐and‐neck plans with the DMLC technique were 5.6% per mm for the CTV‐equivalent uniform dose (EUD).^21^ From the viewpoint of high accuracy of MLC positioning (−0.01 ± 0.02 mm) in this study, the effect of machine control accuracy on the dosimetric accuracy was relatively smaller than other uncertainties. Although this study showed high accuracy of MLC positioning, MLC positions indicated by the log file are considered to contain intrinsic uncertainties. Neal et al. reported that MLC positions derived from the log file differed by approximately 1 mm from actual MLC positions derived from electronic portal imaging device‐based measurements.^21^ However, monitoring MLC position errors during treatment is important for evaluations of dose of the day.^22^, ^23^, ^24^

Maximum dosimetric deviations of CTV D_98%_ and D_2%_ due to residual setup errors ≤2 mm per 2° were 0.5% or less. This study showed that dosimetric errors of CTV were small for residual setup errors (2 mm/2°) since the PTV margin was set to 7 mm. Guckenberger et al. reported that residual setup errors after repositioning with a 6DOF couch were 1.6 ± 0.8 mm with knee support.^13^

Therefore, 2 mm/2° was sufficient to simulate patient residual setup error. The dosimetric errors in the CTV due to setup error were sufficiently small when the patient repositioning with CBCT‐based IGRT was performed and the PTV margin was set to 7 mm. The PTV margin should be considered for methods to correct patient setup errors, repositioning with 6DOF or 3DOF, and bony or tumor matching.^25^

Compared with the small dosimetric errors caused by mechanical control uncertainties and residual setup errors, the errors in the fractional delivered doses led to large underdosing of the CTV D_98%_ for patients 5 and 13 in Fig. 1. These results showed that the CTVs of patients 5 and 13 did not receive the prescription dose during treatment. Therefore, CBCT‐based IGRT by bone matching cannot ensure daily target coverage because of variations in patient weight and deformations of internal organs. Therefore, monitoring delivered doses during the treatment course should be implemented in the clinical workflow to assure the delivered plan quality. Many studies have reported the necessity of adaptive strategies that consider the interfractional variations in bladder and rectum filling.^26^, ^27^, ^28^ However, the dose differences in the CTV D_98%_ for 4 of 13 patients were within 1% during treatment. In Fig. 1, the dose differences in the CTV D_98%_ tended to be underdose in many cases. The effect of tissue edema caused by gynecological chemo‐radiotherapy is considered for the main underdose factor [Figs. 5(a)–5(c)]. On the other hand, gas pockets in the digestive tract causes overdoses [Fig. 5(d)–5(e)]. Although the effects of gas pocket vary from day to day, tissue edema affects dosimetric error over the long term of treatment. Therefore, CTV D_98%_ tended to negative values in many cases in Fig. 1. Specifically, the magnitude of dose differences to a target strongly depends on the patient. Another study by McParland et al.^29^ that evaluated fractional doses to prostate cancers showed that the dose differences to the prostate CTV D_98%_ decreased by 3% for the mean and 4.6% for the maximum. This inconsistency is attributed to the differences in the total treatment period, beam energy, and image quality of CBCT.

In contrast to the evaluations of fractional doses, Fig. 3 shows that the interfractional deformations of internal organs contributed little to the dose differences in the accumulated dose. We found that the factors reducing target coverage could be divided into random and systematic components. The random components were interfractional organ deformation and patient setup errors.

As shown in Fig. 5(a−c), patient 13 showed weight gain during treatment. This variation in patient’s weight caused dose decreases in CTV. In addition, dose increases due to the appearance of the gas pocket in rectum was detected as shown in Fig. 5(d−f). Previous study has also reported the increase in dose due to the gas pocket effect.^30^ These variations of delivered doses are considered as the variation of Total Energy Released per unit Mass (TERMA) and deformations of dose kernel in heterogeneous media. The random presence of the gas pocket causes dose differences from the original plan. In addition, gas pocket influences accuracy of dose calculation based on CBCT images due to artifacts.

To ensure delivery of the prescribed dose for a patient, adaptive radiation therapy (ART) can be considered to prevent or minimize this dosimetric errors caused by patient body weight changes. A previous study discussed the importance of ART for head‐and‐neck cancer.^31^ Although the ART technique was generally focused on head‐and‐neck cancers, the study showed that the ART technique could be applied to the pelvic region. We think that evaluation of fractional delivered doses should be an essential quality assurance technique used during the whole treatment course.

In this study, the HU‐to‐relative electron density (RED) conversion method was applied for CBCT‐based dose calculations. A limitation of this study was the inaccuracy of the CBCT‐based dose calculation since poor image quality and inaccurate HU, compared with those in planning CT, are caused by scatter photons in the patient body and reconstruction techniques. However, Barateau et al. found that the accuracy of the CBCT‐based dose calculation for a pelvic anthropomorphic phantom was <2.4%.^32^ Consequently, the calculation accuracy is sufficient for evaluation of fractional doses but not for replanning. In addition, the advantage of the HU‐to‐RED conversion method is that it is easy to introduce to a clinical workflow. The improvements in image quality of CBCT with machine learning have been recently investigated.^33^, ^34^ Application of this novel technique to evaluate delivered doses will be studied in the future.

Dosimetric uncertainties caused by intrafraction motion are also required to investigate the evaluation of dose delivery to the target. However, since intrafraction organ motions are continuous, the accurate dosimetric evaluation using CBCT which expresses only the imaging moments is substantially difficult. Haripotepornkul et al measured the inter‐ and intrafractional movements of cervix with OBI.^35^ They reported that geometric uncertainty of cervix caused by interfractional motion was larger than that of intrafractional motion. Since the accumulated dose evaluation of CTV D_98%_ indicated that interfractional delivery errors were within 1.7%, the effect of intrafractional motion is considered to be even smaller.

Hence, DIR should be introduced for more accurate evaluation of fractional and accumulated dose differences in tumors and OARs.^36^ Takayama et al. reported the high accuracy of DIR used with a hybrid DIR algorithm that incorporates both contour‐based registration and image intensity‐based registration for prostate cancer patients.^15^ However, in the case of gynecology with the large fields including gastrointestinal tract, artifacts of CBCT images caused by intestinal gas decrease accuracy of dose calculation and deformable image registration.

Patients 2 and 8 instructed the bladder and rectum to be emptied before planning CT and treatment. However, compared to patient 8, patient 2 showed a larger difference from the planned dose [seen in Fig. 4(a)]. Patient 2 also had large dosimetric errors of CTV in Figs. 1 and 2. Figure 4 and Table 3 indicate that reproducibility of organ volume and gas pockets varies from patient to patient. Although the deformed accumulation dose was evaluated only for two patients without artifact in all fractions, as shown in Table 2, deformation accuracy of hybrid DIR was smaller than previous study.^15^ To improve accuracy of evaluation for accumulated delivered doses of OARs, generating synthesized CT image based on CBCT by a machine learning algorithm^33^, ^34^ will be introduced for reducing artifact effects in future work. Then, actual delivered doses of OARs will be analyzed with synthesized CT generated by CBCT and hybrid DIR technique in next step.

The same trend was found in dosimetric errors of CTV accumulated by rigid registration (Figs. 1 and 2) and dosimetric errors of CTV and OARs accumulated by DIR (Fig. 4). Then, this study shows the possibility of evaluating accumulated dosimetric errors of CTV and OARs by fractional delivered dose error of CTV with commercial software. When fractional delivered dose errors of CTV D_98%_ exceeds 2%, ART should be required to ensure delivery accuracy of CTV and OARs. ART can eliminate the effects of dosimetry on patient weight changes and gas pocket fluctuations in planning CT and treatment.

Kershaw L et al reported that position errors of regional lymph nodes were inconsistent with bone matching.^25^ Although the residual setup errors in bone matching were different for tumor and lymph node, these setup errors were sufficiently covered by PTV margin.

This work clarified that our institution could sufficiently deliver prescription dose to CTV. Although enough margin was added to CTV, interfractional variations of patient weight changes and gas pocket caused large dose differences (~5%) seen in Fig. 5.

Not only expanding PTV margin, adaptive radiation therapy will be required to eliminate these factors related to daily patient physiological changes. Jensen et al reported feasibility of adaptive strategy for cervical cancer therapy by daily CBCT‐based monitoring by radiation therapists.^37^ This study showed that monitoring fractional delivered doses helps radiation oncologists and medical physicists in decision making of replanning.

In conclusion, three categories of factors that contribute to decreased dose delivery accuracy were evaluated in this study. We found that patient weight variations and internal organ deformations caused large target dose differences from the original plan doses. Monitoring of delivered doses should be added to a clinical workflow to periodically evaluate the delivered plan quality.

## CONFLICT OF INTEREST

There is no conflict of interest related to this study.

## AUTHOR CONTRIBUTION STATEMENT

All co‐authors revised the manuscript. Yohei Utena collected and analyzed the data. Yohei Utena and Jun Takatsu prepared the manuscript. Jun Takatsu is the corresponding author and designed the study. Satoru Sugimoto provided supervision of the manuscript. Keisuke Sasai provided final approval of the manuscript.

## Funding information

This study was not funded.
